# Molecular cloning of a novel *bioH* gene from an environmental metagenome encoding a carboxylesterase with exceptional tolerance to organic solvents

**DOI:** 10.1186/1472-6750-13-13

**Published:** 2013-02-15

**Authors:** Yuping Shi, Yingjie Pan, Bailin Li, Wei He, Qunxin She, Lanming Chen

**Affiliations:** 1 Key Laboratory of Quality and Safety Risk Assessment for Aquatic Products on Storage and Preservation (Shanghai), China Ministry of Agriculture, Engineering Centre for Quality Control and Risk Assessment of Aquatic Products, College of Food Science and Technology, Shanghai Ocean University, 999 Hu Cheng Huan Road, 201306, Shanghai, P. R. China; 2 Shanghai Hanyu Bio-lab, 151 Ke Yuan Road, 201203, Shanghai, P.R. China; 3 Department of Biology, University of Copenhagen, Ole Maaloes Vej 5, 2200 N, Copenhagen, Denmark

**Keywords:** BioH, Biotin biosynthetic pathway, Carboxylesterase, Metagenome, Aqueous environment

## Abstract

**Background:**

BioH is one of the key enzymes to produce the precursor pimeloyl-ACP to initiate biotin biosynthesis *de novo* in bacteria. To date, very few *bioH* genes have been characterized. In this study, we cloned and identified a novel *bioH* gene, *bioHx*, from an environmental metagenome by a functional metagenomic approach. The *bioHx* gene, encoding an enzyme that is capable of hydrolysis of *p*-nitrophenyl esters of fatty acids, was expressed in *Escherichia coli* BL21 using the pET expression system. The biochemical property of the purified BioHx protein was also investigated.

**Results:**

Screening of an unamplified metagenomic library with a tributyrin-containing medium led to the isolation of a clone exhibiting lipolytic activity. This clone carried a 4,570-bp DNA fragment encoding for six genes, designated *bioF*, *bioHx*, *fabG*, *bioC, orf5* and *sdh*, four of which were implicated in the *de novo* biotin biosynthesis. The *bioHx* gene encodes a protein of 259 aa with a calculated molecular mass of 28.60 kDa, displaying 24-39% amino acid sequence identity to a few characterized bacterial BioH enzymes. It contains a pentapeptide motif (Gly_76_-Trp_77_-Ser_78_-Met_79_-Gly_80_) and a catalytic triad (Ser_78_-His_230_-Asp_202_), both of which are characteristic for lipolytic enzymes. BioHx was expressed as a recombinant protein and characterized. The purified BioHx protein displayed carboxylesterase activity, and it was most active on *p*-nitrophenyl esters of fatty acids substrate with a short acyl chain (C4). Comparing BioHx with other known BioH proteins revealed interesting diversity in their sensitivity to ionic and nonionic detergents and organic solvents, and BioHx exhibited exceptional resistance to organic solvents, being the most tolerant one amongst all known BioH enzymes. This ascribed BioHx as a novel carboxylesterase with a strong potential in industrial applications.

**Conclusions:**

This study constituted the first investigation of a novel *bioHx* gene in a biotin biosynthetic gene cluster cloned from an environmental metagenome. The *bioHx* gene was successfully cloned, expressed and characterized. The results demonstrated that BioHx is a novel carboxylesterase, displaying distinct biochemical properties with strong application potential in industry. Our results also provided the evidence for the effectiveness of functional metagenomic approach for identifying novel *bioH* genes from complex ecosystem.

## Background

Biotin (also known as vitamin H, or B7) is an essential enzyme cofactor involved in multiple important metabolic pathways including fatty acids, amino acids, and carbohydrates in all three domains of life [1]. This co-enzyme is synthesized *de novo* in microorganisms [2] where enzymes catalyzing the last four steps in the biotin biosynthesis are well characterized. These include 7-keto-8-aminopelargonic acid (KAPA) synthase, 7, 8-diaminopelargonic acid synthase, dethiobiotin synthetase, and biotin synthase. Their encoding genes, designated *bioF*, *bioA*, *bioD* and *bioB*, have been identified in *E. coli*[2,3]. More recently, two pathways, BioC-BioH and BioI, have been suggested for synthesizing pimeloyl-ACP (acyl carrier protein), an efficient substrate of BioF [2]. The BioI pathway is only present in *Bacillus subtilis* and its close relatives, where pimeloyl-ACP is to be synthesized by an O_2_-dependent cleavage of long chain acyl-ACPs [2,4]. In the BioC-BioH pathway, BioC catalyzes S-adenosylmethionine-dependent methylation of malonyl-CoA (or ACP) to malonyl-CoA (or ACP) methyl ester that enters fatty acid synthetic pathway, where an intermediate pimeloyl-ACP methyl ester was cleaved by BioH to produce pimeloyl-ACP that serves as the substrate for BioF to initiate biotin synthesis [2,5]. To date, *bioH* genes have been cloned from *E. coli*, *Serratia marcescens, Serratia* sp. and *Kurthia* sp. strains, and the encoded proteins exhibit carboxylesterase activity [6-9].

Esterases (EC 3.1.1.1) catalyze the hydrolysis of esters with short-chain fatty acids. They belong to the hydrolase superfamily with α/β protein fold [10], carrying a serine nucleophile embedded in the pentapeptide motif Gly-Xaa-Ser-Xaa-Gly [11] and the catalytic triad Ser-His-Asp [12] at the catalytic site. Esterases, including lipases, are very important industrial enzymes and are widely applied in detergents and degreasing formulations, paper and leather manufacture, food processing, medical diagnostics and pharmaceutical syntheses, as well as wastewater treatment [13]. Microorganisms comprise the main resources of hydrolases from which most commercial hydrolases are derived [13]. This is mainly because microbes exhibit much great diversity than multicellular organisms. As a result, screening for novel hydrolases from microbe-rich environments have been of a great interest from both industrial and academic standpoints.

Since up to 99% of microorganisms in environments cannot be cultured by standard cultivation techniques according to taxonomic studies based on 16S rRNA genes [14,15], exploring esterase resources with any culture-dependent methodology renders those resources untapped for uncultured microbes. Recently metagenomic technology was developed to address this problem. Utilizing this culture-independent approach, several genes encoding novel industrial enzymes, including lipases and esterases, have been obtained [16-19]. However, to our knowledge, hydrolase resources have not been explored for microbial communities in aquaculture environments, such as fishing pond sediments where diverse microorganisms thrive according to 16S rRNA gene analysis (unpublished data). Here we report the cloning and identification of a *bioH* gene coding for an enzyme that is capable of hydrolysis of *p*-nitrophenyl (*p*NP) esters of fatty acids by a functional metagenomic approach. A novel *bioH*, *bioHx*, was expressed, and the recombinant protein was purified and characterized. Our results indicate that BioHx is a novel carboxylesterase involved in the biotin biosynthesis, exhibiting good application potential in industry and biotechnological research.

## Results and discussion

### Identification of a tributyrin-hydrolyzing clone from a metagenomic library and sequence analysis of the cloned DNA fragment

Metagenomic DNA was prepared from sediment samples of aquaculture ponds. The resultant DNA were mechanically sheared by sonication, yielding DNA fragments of 2-9 kb that were used to construct a metagenomic library as described in Methods. To test the quality of the library, twenty transformants grown on LB-ampicillin agar plates were randomly picked up for plasmid preparation. Digestion of the purified plasmids with *Eco*RI indicated that they all contained an insert DNA fragment of 2-6 kb (data not shown), indicating the library was suitable for functional screening of industrial enzymes. In an esterase screening with Spirit Blue Agar plates containing tributyrin, one clone was found to produce a clear zone of hydrolysis on the selective plate amongst ca. 9,000 transformants yielded from the metagenomic library. This clone, designated pEstbioHx, was used for further studies.

**Figure 1:**
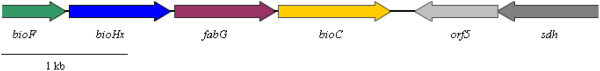
**Gene organization of the sequenced 4,570-bp fragment cloned from an environmental metagenomic library.** Six ORFs were identified, including: *bioF,* encoding 7-keto-8-aminopelargonic acid (KAPA) synthase; *bioHx*, encoding carboxylesterase; *fabG*, encoding 3-ketopimeloyl-ACP methyl ester reductase; *bioC*, encoding S-adenosylmethionine-dependent methyltransferase; *orf5*, encoding a DUF218 domain protein of unknown function; and *sdh*, encoding serine/threonine dehydratase.

The complete DNA sequence of the cloned fragment in pEstbioHx was determined, and deposited in the GenBank sequence database under the accession no. JX870139. It is 4,570-bp in length and contains six genes, designated *bioF*, *bioHx*, *fabG*, *bioC, orf5* and *sdh*. Their gene organization is presented in Figure 1, in which the genes at the two ends were only recovered partly, i.e. the 5´ end of *bioF* and 3´end of *sdh*. Database searches revealed that the 4.5-kb sequence displayed 91% sequence identity at the nucleotide level over its full length with a DNA fragment of the *Stenotrophomonas maltophilia* R551 genome [GenBank: CP00111.1], indicating that the cloned DNA fragment could be derived from a *S. maltophilia* strain present in the fishing pond sediments. This is consistent to the notion that *S. maltophilia* is ubiquitous in aqueous environments, soil and plants [20].

**Figure 2:**
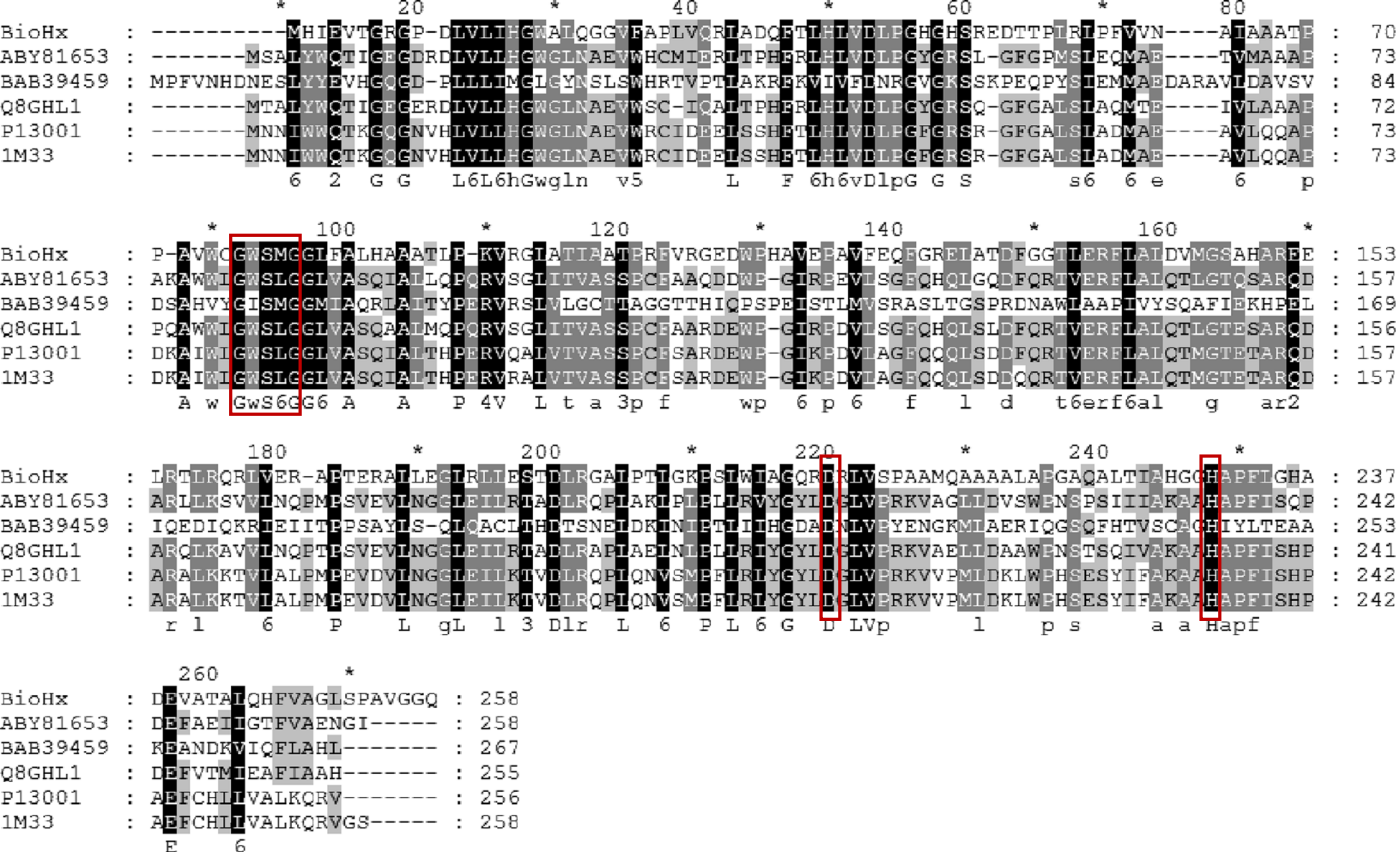
**A multiple sequence alignment of BioHx and other known bacterial BioH proteins.** The numbering of sequences is indicated above the alignments. The starting and ending residue numbers of each protein sequence used in this study are indicated before and after each sequence. Identical amino acid residues as well as the conserved ones (>50% of the sequences) are highlighted in black and in grey, respectively, with the consensus sequence shown below each alignment. The pentapeptide motif and catalytic triad are shown in boxes. The BioH sequences chosen for the analysis are from the GenBank database, except 1M33 from the PDB database: ABY81653, *Serratia sp.* SES-01; BAB39459, *Kurthia sp.* 538-KA26; Q8GHL1, *Serratia marcescens* Sr41; P13001, *Escherichia coli* K-12; 1M33, *Escherichia coli* DH5α; BioHx, obtained in this study.

The gene responsible for the hydrolytic activity of tributyrin, was identified as *bioHx* positioned from 518 to 1297 in the cloned DNA fragment. *bioHx* encodes a protein of 259 aa, displaying high amino acid sequence similarities to putative carboxylesterase BioH identified from the complete genomes of *Stenotrophomonas* (95-97% identity) and *Xanthomonas* (70-73% identity) species in the public databases. Much lower sequence similarities were found between BioHx and BioH enzymes of *Escherichia coli* K12 (38% identity in a 233 aa stretch, [GenBank: P13001]) [6], *Escherichia coli* DH5α (37% identity in a 242 aa stretch, [PDB: 1M33]) [12], *Serratia* sp. SES-01 (38% identity in a 250 aa stretch, [GenBank: ABY81653]) [6], *Serratia marcescens* Sr41 (39% identity in a 250 aa stretch, [GenBank: Q8GHL1]) [7], and *Kurthia sp.* 538-KA26 (24% in a 256 aa stretch, [GenBank: BAB39459]) [9], the only bacterial BioH that have been cloned and available in the databases. Analysis of these BioH proteins by multiple sequence alignment revealed that both the pentapeptide motif Gly_76_-Trp_77_-Ser_78_-Met_79_-Gly_80_ and the catalytic triad Ser_78_-His_230_-Asp_202_ are conserved in BioHx (Figure 2).

As illustrated in Figure 1, the first 4 genes encoded in the cloned DNA fragment could form an operon with other biotin synthetic genes. The incomplete *bioF* gene (<1-500 nt) is located upstream of *BioHx*, coding for a KAPA synthetase protein with an N-terminal truncation. BioF is the enzyme catalyzing the first of the four-step reactions in biotin biosynthetic pathway. It synthesizes KAPA with pimeloyl-ACP and L-alanine [3]. The BioF displayed high sequence identities at the amino acid level to the same protein encoded in different bacteria including *Stenotrophomonas* (90-97%) and *Xanthomonas* (69-75%), as well as other bacteria, such as *Xylella*, *Allochromoatium* and *Pseudomonas* (51-69%). There is a noncoding region of 17 bp separating *bioF* from *bioHx* but it does not carry any recognizable motif for a putative promoter or a ribosomal binding site. Downstream of the *bioHx* lay *fabG* (1327-2103 nt) and *bioC* (2119-2985 nt) genes. The *bioC* encodes a protein of 294 amino acids, showing a high sequence similarity to a putative biotin biosynthesis protein BioC from *Stenotrophomanas* and close related species, while the deduced amino acid sequence of the 777-bp *fabG* gene exhibits a high level of sequence identity to putative 3-ketopimeloyl-ACP methyl ester reductases involved in fatty acid biosynthesis in the database. FabG reduces 3-ketopimeloyl-ACP methyl ester to 3-hydroxypimeloyl-ACP methyl ester in canonical fatty acid biosynthetic pathway, via which the intermediate pimeloyl-ACP methyl ester was produced and hydrolyzed by BioH to form pimeloyl-ACP substrate for BioF [3]. The intergenic regions are only for 29 and 15-bp starting from the second gene through the fourth with no identifiable putative promoters. Thus, *bioF, bioHx, fabG* and *bioC* could be co-transcripted. Several other bacteria carry the same gene organization in the *bioH* region as for the cloned metagenomic fragment in which *bioH* is a member of the biotin biosynthetic operon [21]. But *bioH* genes in *E. coli* K12 and *Serratia* sp. SES-01 (denoted as *bioHe* and *bioHs*, respectively) are located elsewhere in the genomes [6]. Taken together, this reveals an interesting aspect of bacterial *bioH* diversity and evolution.

Two additional genes on the sequenced DNA fragment, *orf5* (3798-3166 nt) and *sdh* (4570-3795 nt) could have different functions. They are in the opposite orientation as for the first four genes. *orf5* encodes a protein of 210 aa, and its closest match was a protein family containing DUF218 domain. However, there is no clue that could lead to a possible function for this protein family in the current literature. The N-terminal truncated Sdh protein displayed a high degree of similarity with serine/threonine dehydratase, which functions in bacterial amino acid metabolism. As there is 3 bp overlap between the coding sequences of *orf5* and *sdh* genes, they could form an operon together with other unrecovered genes. It is thus interesting to study if *orf5* could have any functional connection to *sdh*.

**Figure 3:**
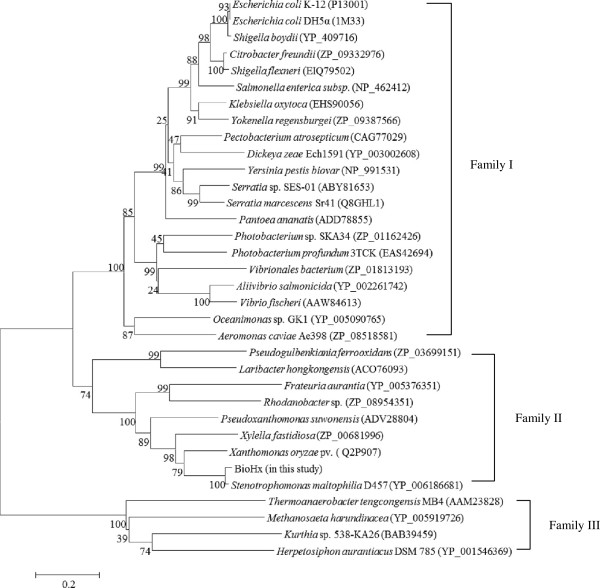
**A phylogenetic tree showing evolutionary relationship between BioHx and a related set of BioH proteins identified in the public databases.** The neighbor-joining phylogenetic tree was constructed using the software MEGA 4.0. Bootstrap percentages are shown at nodes. The scale bar represents 0.2 changes per amino acid.

Based on the deduced amino acid sequences of BioHx and a selected set of its homologs identified in the public databases, a phylogenetic tree was constructed by the MEGA4.0. This analysis revealed that these BioH sequences could form three families, designated I, II, and III (Figure 3). The BioH identified from *E. coli*, *Serratia* sp. and *S. marcescens*, as well as *Kurthia* sp. [6-9] were distributed in family I and III, respectively, whereas BioHx cloned in this study fell into cluster II, representing a novel BioH.

### Expression and purification of *bioHx* gene product

The 777-bp *bioHx* structural gene (not including the stop codon) was amplified with primers BioHx-MF and BioHx-MR, the purified PCR product was digested with *Bam*HI and *Hind*III, and then cloned into the expression vector pET-28a at *Bam*HI and *Hind*III sites. The yielded recombinant plasmid, pET28a-*bioHx*, was transformed into *E. coli* BL21. Kan^r^ transformants containing pET28a-*bioHx* were screened by colony PCR. *Bam*HI and *Hind*III digests of the pET28a-*bioHx* verified that it carried the *bioHx* structural gene (data not shown).

**Figure 4:**
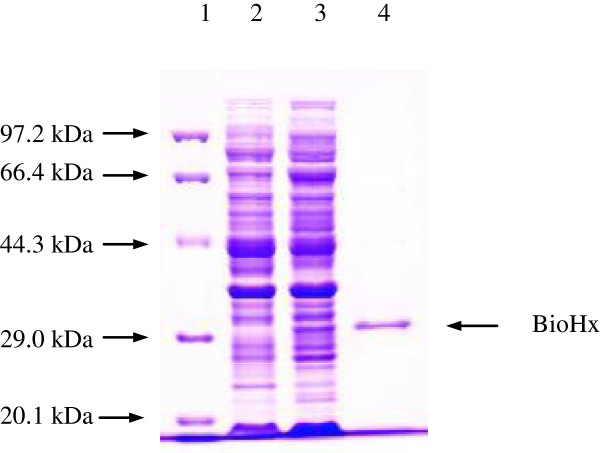
**SDS-PAGE analysis of the *bioHx *gene product.** Protein molecular weight marker (Tiangen, Cat. No. DPP530S) (Lane 1), soluble proteins of *E. coli* BL21 (pET28a) (Lane 2) and *E. coli* BL21 (pET28a-*bioHx*) (Lane 3) cultured and induced under the same condition at 21°C for 20 h, and purified recombinant BioHx (29.27 kDa) (Lane 4).

A pET28a-*bioHx* transformant was grown in LB-kanamycin medium. The expression of BioHx was induced by adding 0.6 mM IPTG to the culture, and the induction was at 21°C for 20 h. The cell-free extract containing the soluble target protein was analyzed by SDS-PAGE. As presented in Figure 4 (lane 3), one band corresponded in size to the calculated molecular mass of BioHx was detected. The band was absent in control lane from the *E. coli* BL21 cells carrying only the non-recombinant pET28a vector (Figure 4, lane 2), which was cultured and induced under the same condition as that for the *E. coli* BL21 (pET28a-*bioHx*).

The expressed recombinant protein with six his-tags at C-terminal was purified via a Ni-NTA His Bind resin column. Figure 4 (lane 4) shows the purified BioHx with molecular mass of approximately 29.27 kDa. Purified protein was about 82.70 mg of per 1 g wet biomass, and accounted for about 1.8% of soluble total proteins in the cell-free extract.

### Effect of temperature and pH on BioHx activity and stability

**Figure 5:**
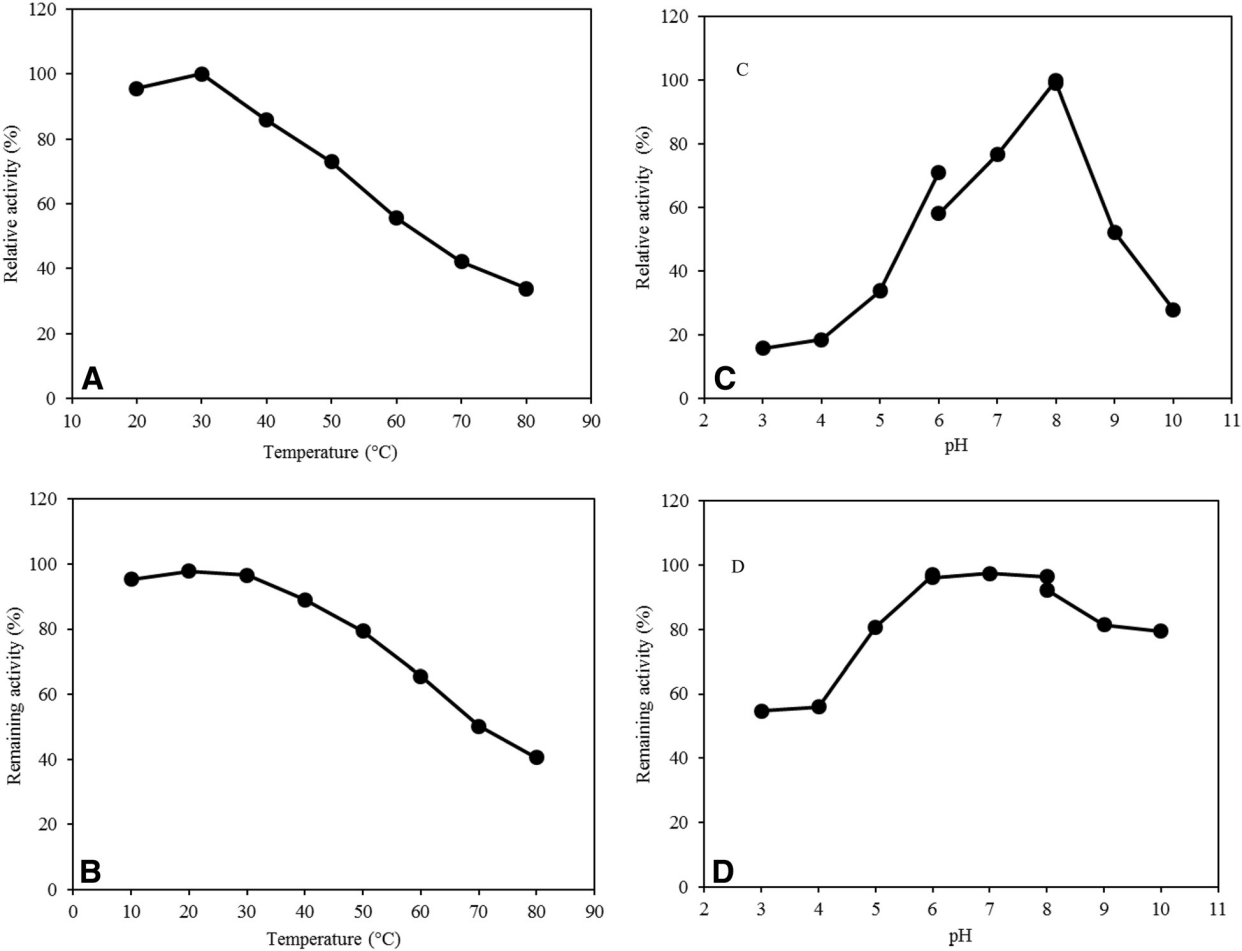
**Effect of temperature and pH on BioHx activity and stability.** Relative activity of purified BioHx was determined at different temperatures (**A**) or pH (**C**) using pNP-butyrate as the substrate at 405 nm. Remaining enzyme activity was measured at 30°C and pH 8.0 after incubating purified BioHx at different temperatures for 30 min (**B**), or pH at 4°C for 12 h (**D**). All determinants were performed in triplicate.

Carboxylesterase activity of the purified BioHx was determined for temperature range of 20 to 80°C using pNP-butyrate as the substrate. As presented in Figure 5A, BioHx displayed the maximal enzyme activity at 30°C. The enzyme exhibited more than 74% of the optimal specific activity for the temperature range of 20-50°C, indicating that it is moderately thermotolerant. However, enzyme activity declined rapidly when reaction temperatures reached above 70°C. These results indicated that bioHx is a mesophilic enzyme.

To assess the effect of temperature on BioHx stability, the enzyme was exposed to a broad range of temperature for over 30 min, and the residual activity was measured. As shown in Figure 5B, BioHx showed considerable stability and retained 83-97% of its initial activity after the treatment at 10-50°C. In addition, no loss in activity was observed after the enzyme was stored at 4°C for 12 h (data not shown).

The effect of pH on the BioHx activity was determined at 30°C, the optimal reaction temperature. Purified BioHx was found to function in a range of pH 7.0–9.0 with more than 50% of its maximal activity, and the pH optimum was recorded as pH 8.0 (Figure 5C). Relative activity of BioHx declined sharply at the acidic range of pH. The enzyme stability at various pH values was measured after incubating BioHx at 4°C for 12 h. As shown in Figure 5D, the enzyme exhibited relatively strong stability at pH values ranging from neutral to alkaline conditions. It retained the maximal activity in the pH range of 6.0 to 8.0, which dropped only slightly at pH 10, indicating that the enzyme has a remarkable stability under alkaline conditions.

### Substrate specification of BioHx

**Figure 6:**
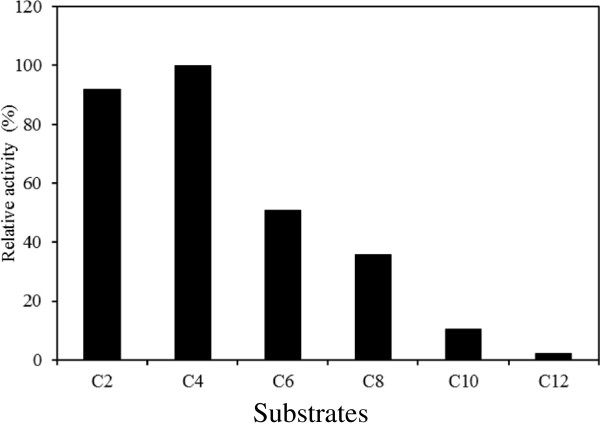
**Substrate specificity of purified BioHx on *p *-nitrophenyl esters of fatty acids.** C2, *p*NP-acetate; C3, *p*NP-propionate; C4, *p*NP-butyrate; C6, *p*NP-caproate; C8, *p*NP-caprylate; C10, *p*NP-caprate; C12, *p*NP-laurate. Relative activity was shown as compared with the activity on C4. All determinants were performed in triplicate.

Carboxylesterase activity of BioHx on *p*-nitrophenyl esters with acyl chain length of up to 12 carbons was assayed at 30°C and pH 8.0. As presented in Figure 6, BioHx displays highest activity (2.7 +/− 0.3 μmol es/ min · mg protein) on *p*NP- butyrate (C4), followed by 89% relative activity on *p*NP-C2, and 39-52% on *p*NP-C6~C8. These results are in a good agreement with those obtained with other bacterial BioH enzymes [6,12]. At structural level, the catalytic triad of *E. coli* BioH [PDB: 1M33] is buried between two domains and was not readily accessible for bulkier compounds [12]. This serves as a good explanation as to why BioH enzymes are more active on substrates with a short side chain.

It was reported that BioH encoded in *E. coli* showed a low enzymatic activity of thioesterase using palmitoyl-CoA as a substrate [12]. Given that BioHx contained Gly_76_-Trp_77_-Ser_78_-Met_79_-Gly_80,_ a conserved motif for acyltransferases and thioesterases, we studied the thioesterase activity of BioHx. The assay was carried out at 30°C and pH 8.0 using palmitoyl-CoA as the substrate. BioHx showed a specific activity of 0.20 +/− 0.08 μmol CoA/min · mg protein on palmitoyl-CoA, which was approximately 13.4-times lower than its carboxylesterase activity on pNP-butyrate. The ratio of BioHx activities of hydrolyzing *p*NP-esters/CoA-thioester was similar to that of BioHs (11.6:1), but different from that of BioHe (33.3:1) [6].

### Effect of metal ions on BioHx stability

**Figure 7:**
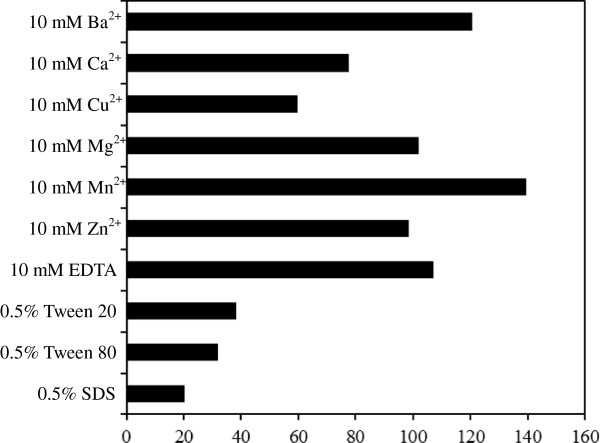
**Effect of metal ions and detergents on BioHx Stability.** Aliquots of the enzyme were incubated with each metal ion (10 mM) and detergent (0.5%) at 30°C for 1 h, the residual activity of BioHx was determined using pNP-butyrate as the substrate. All determinants were performed in triplicate.

To test the effect of divalent metal ions on BioHx activity, aliquots of the enzyme was incubated with 10 mM of ZnCl_2_, MnCl_2_, CaCl_2_, MgCl_2_, BaCl_2_, and CuSO_4_ at 30°C for 1 h, and residual activity was measured at 30°C and pH 8.0 using pNP-butyrate as the substrate. As presented in Figure 7, compared with the control, Ba^2+^ and Mn^2+^ were found to increase enzyme activity by 20.5 and 39.2%, respectively, whereas Ca^2+^ and Cu^2+^ showed a moderate inhibition to the enzyme as the activity declined to 77.7 and 59.5%, respectively. Mg^2+^ and Zn^2+^ did not affect the enzyme activity. EDTA (10 mM) showed no effect on BioHx activity, suggesting that it is not a metalloenzyme. This is consistent with the results obtained with BioHe and BioHs [6].

### Effect of detergents and organic solvents on BioHx stability

The effect of ionic and nonionic detergents on BioHx stability including sodium dodecyl sulfate (SDS), Tween 20 and Tween 80 was assayed. Similar with BioHe and BioHs, SDS strongly inhibited BioHx activity, diminishing about 80% of its activity at 0.5% concentration (Figure 7). In contrast to BioHe and BioHs in which their activities were barely affected by 0.2-5% of Tween 20 and Tween 80 [6], the non-ionic detergents greatly reduced the BioHx activity, inhibiting more than 60% of its activity at a final concentration of 0.5%, arguing for the diversity of BioH enzymes.

Tolerance to organic solvents is very important for any enzymes to be explored for industrial application. To study the stability of BioHx in organic solvents, the enzyme was individually incubated with methanol, ethanol, 2-propanol, 1-butanol, acetonitrile, acetone, dimethyl sulfoxide (DMSO) and dimethylformamide (DMF) at the indicated concentrations at 30°C for 1 h. Residual activity of the treated enzymes was then determined under the standard assay condition. As summarized in Table 1, BioHx exhibited remarkable stability in methanol, DMSO and DMF, as it retained more than 90% of the original activity after the treatment with each of the organic solvents at a final concentration of 30%. Organic solvent tolerance has been reported for BioHe and BioHs, from which different results have been obtained. BioHe shows strong resistance to treatments with 30% organic solvents, whereas BioHs lost more than 95% activity under the same treatments [6]. However, compared with BioHe, BioHx is more resistant to the treatments with 30% of 2-propanol or acetonitrile, as the residual activity of the treated BioHx is ca. 3-fold and 7-fold of that for BioHe. Furthermore, upon treatments with 50% 2-propanol or acetonitrile, BioHx still exhibits 76.2 or 44.3% residual activity (Table 1), indicating that it is very resistant to these organic solvents. These results reinforce that BioHx represents a novel BioH enzyme that is very suitable to be exploited for commercial application.

**Table 1:** Effect of organic solvents on BioHx stability after incubation at 30°C for 1 h

**Organic solvents**	**Residual activity (%)**			
	**20%**	**30%**	**40%**	**50%**
Control	100 ± 0.10	100 ± 0.00	100 ± 0.00	100 ± 0.00
Methanol	94.6 ± 3.2	93.6 ± 4.4	85.5 ± 2.3	57.0 ± 4.0
Ethanol	85.5 ± 0.8	82.2 ± 3.2	77.9 ± 2.1	75.4 ± 4.1
2-Propanol	86.3 ± 3.2	86.3 ± 4.5	76.8 ± 4.4	76.2 ± 3.0
1-Butanol	48.9 ± 3.6	47.9 ± 3.0	30.8 ± 2.7	23.5 ± 2.2
Acetonitrile	74.4 ± 1.2	74.4 ± 3.3	65.0 ± 4.3	44.3 ± 4.5
Acetone	79.6 ± 1.9	78.0 ± 2.7	62.1 ± 3.7	15.9 ± 4.4
DMSO	95.7 ± 2.4	95.3 ± 1.9	54.2 ± 1.0	29.7 ± 3.8
DMF	97.7 ± 0.8	97.3 ± 1.3	72.3 ± 2.1	19.7 ± 1.1

No organic solvent was included in the control samples. The concentrations of organic solvents are indicated (v/v). The enzyme activity was determined under the standard assay condition, and the data is represented as an average of three independent determinations.

Although *bioH* genes are present in many microbial genomes in the public databases, very few BioH enzymes have been biochemically characterized to date. Among them, interesting diversity of BioH enzymes is emerging, such as the great differences in organic solvent tolerance revealed for BioHe, BioHs and BioHx. In this regard, more BioH enzymes should be investigated both for elucidating biotin biosynthetic mechanisms in microorganisms and for exploiting them for commercial application in ester hydrolysis. There are three alternative approaches to select *bioH* genes for biochemical characterization: (1) identifying *bioH* genes from the public databases, (2) isolating microorganisms from environments for studying *bioH*, or (3) functional screening for *bioH*-containing clones from metagenomic libraries. Since functional screening of metagenomic libraries explore all microbes in environments where more than 90% of the resources are not represented in any culture-dependent approach and far more less resources are included in the public databases, we regard that the metagenomic approach represents the most rewarding research to explore industrial application of BioH enzymes.

## Conclusions

In this study, a novel *bioH* gene, *bioHx,* in a 4,570-bp DNA fragment containing a biotin biosynthetic gene cluster was cloned and identified from an environmental metagenome by a functional metagenomic approach. The *bioHx* gene encodes a protein of 259 aa with a calculated molecular mass of 28.60 kDa, displaying 24-39% amino acid sequence identity to a few characterized bacterial BioH enzymes. BioHx was expressed as a recombinant protein in *Escherichia coli* BL21 using the pET expression system. The purified BioHx protein displayed carboxylesterase activity, and it was most active on *p*-nitrophenyl esters of fatty acids substrate with a short acyl chain (C4). Comparing BioHx with other known BioH proteins revealed interesting diversity in their sensitivity to ionic and nonionic detergents and organic solvents, and BioHx exhibited exceptional resistance to organic solvents, being the most tolerant one amongst all known BioH enzymes. The distinct bio-chemical properties positioned BioHx as a novel carboxylesterase with a good application potential in industry and biotechnological research.

## Methods

### Bacterial strains, plasmids and culture conditions

*Escherichia coli* TOP10 [genotype: F¯ *mcr*A **Δ**(*mrr*-*hsd*RMS-*mcr*BC) Φ80 *lac*Z **Δ**M15 **Δ***lac*X74 *rec*A1 *ara*D139 **Δ**(*ara*-*leu*)7697 *gal*U *gal*K *rps*L (Str^R^) *end*A1-*nup*G] (TianGen Biotech Co. Ltd. Beijing, China) was used as a host strain for transformation of recombinant plasmids in the cloning. *E. coli* BL21(DE3) [genotype: F^-^*omp*T *hsd* S_B_(*r*_B_^-^*m*_B_^-^) *gal dcm*(DE3)] (TianGen) was employed as a host for expression of recombinant proteins. The plasmid pUC19 (Fermentas, MD, USA) and pET-28a (Merck Millipore, Darmstadt, Germany) were used as cloning and gene expression vector, respectively. *E. coli* host cells were grown in Luria-Bertani (LB) liquid medium [22] at 37°C with shaking at 200 rpm, while *E. coli* strains containing recombinant plasmids were cultured in LB broth or agar plates supplemented with ampicillin (100 μg/ml) or kanamycin (30 μg/ml).

### Extraction of metagenomic DNA from sediment in aquaculture ponds

Sediment samples were collected from a few aquaculture ponds farming shrimp (*Litopenaeus vannamei*) located in Shanghai, China. Extraction of metagenomic DNA was carried out immediately after samples were transported to our laboratory in Shanghai Ocean University (Shanghai, China) according to the method of *in situ* lysis of microorganisms described by Zhou et al. [23]. A sediment sample (50 g) and 135 ml DNA extraction buffer [100 mmol/l Tris-HC1, 100 mmol/l EDTA, 1.5 mol/l NaC1 and 1% (w/v) hexadecyltrimethylammonium bromide (Sigma-Aldrich, MO, USA), pH8.0] were blended vigorously at 37°C for 30 min. Then 0.5 ml protease K (20 mg/ml) and 2.0 ml SDS (20%) were added, and the mixture was incubated at 50°C for 3 h with gentle end-over-end inversions every 20 min. After centrifugation at 6,000 g for 10 min at 25°C, the supernatant was collected and mixed with an equal volume of chloroform: isopropyl alcohol (24:1, by vol). The upper aqueous phase containing DNA was recovered by centrifugation at 12,000 g at 4°C for 10 min, and DNA was precipitated by ethanol precipitation using 0.1 volume of 3M sodium acetate (pH 5.3) and 2 volumes of chilled anhydrous ethanol. To enhance the rate of DNA recovery, the mixed solution was held at -20°C for 2 h, and centrifugated at 13,000 g for 15 min at 4°C. DNA pellet was washed with 70% ethanol, and resuspended in 200 μl sterilized Mili-Q water (Millipore Billerica, MA, USA). The isolated DNA was further purified using Wizard DNA Clean-Up System (Promega, WI, USA) according to the manufacturer’s instruction. The concentration of DNA in the samples was determined using a multi-mode microplate reader BioTek Synergy™ 2 (BioTek Instruments, Inc., VT, USA).

### Construction of metagenomic library and screening for tributyrin-hydrolyzing clones

Purified metagenomic DNA was sheared mechanically by sonication for appropriate lengths of time established by pilot experiments to generate 2-9 kb DNA fragments. The fragments were separated by agarose gel electrophoresis and recovered from the gel matrix using Axygen gel extraction kit (Axygen, CA, USA). The purified fragments were blunted at 5´ and 3´ends with T4 DNA polymerase and DNA Polymerase I (Large Klenow Fragment) (New England BioLabs, MA, USA), and then ligated to a pUC19 vector at *sma*I site according to the instructions of the manufactures. Ligation was preformed in a 10 μl reaction volume with a molar ratio of vector: insert of 1:3, and incubated at 16°C overnight, yielding an unamplified metagenomic library. This was then used to transform *E. coli* TOP 10 competent cells via the heat-shock method [22]. A 100 μl of transformed cell suspension was spread onto selective Spirit Blue Agar (Sigma-Aldrich) plates containing 1% tributyrin (Sigma-Aldrich), 0.05% Tween 80 (Sigma-Aldrich) and 100 μg/ml ampicillin. Plates were incubated at 37°C for 48 h, and white colonies with a clear zone of hydrolysis were candidates for identifying esterase genes. Library quality was assessed by spreading 100 μl of transformation culture onto LB-ampicillin agar plate containing 40 μl 5-bromo-4-chloro-3-indolyl-β-D-galactopyran-oside (X-gal, 20 mg/ml), and 16 μl isopropyl-β-D-thiogalactopyranoside (IPTG, 50 mg/ml). Plates were incubated at 37°C overnight, and white colonies were randomly selected for further analysis.

### Sequence analyses

Plasmid DNA of positive library clones was isolated using MiniBEST plasmid purification kit (TaKaRa Biotechnology Co. Ltd. Dalian, China). Automated DNA sequencing was carried out using ABI 3730XL capillary sequencer (Applied Biosystems, CA, USA) and BigDye® terminator version 3.1 cycle sequencing kit (Perkin-Elmer, MA, USA) at the China Human Genome Centre (Shanghai, China). Oligonucleotide primers were synthesized by Shanghai Sangon Biological Engineering Technology and Services Co., Ltd. (Shanghai, China).

Sequencing reads were assembled using the ContigExpress software (http://www.contigexpress.com/). Putative functions were inferred using the Basic Local Alignment Search Tool (BLAST) (http://www.ncbi.nlm.nih.gov/BLAST) and ORF finder (http://www.ncbi.nlm.nih.gov/gorf). Multiple sequence alignments were performed using the ClustalW2 software (http://www.ebi.ac.uk/Tools/msa/clustalw2/) [24]. The neighbor-joining method in the molecular evolutionary genetic analysis software package MEGA (version 4.0) [25] was used to construct a phylogenetic tree. A bootstrap analysis with 1000 replicates was carried out to check the reliability of the tree.

### Expression of *bioHx* in *E. coli* and purification of the recombinant protein

Based on the sequence obtained in this study, specific primers BioHx-MF (5´- CGCGGATCCATGCATATTGAAGT-3´) and BioHx-MR (5´-CCCAAGCTTTTGTCCGCCA-3´) targeting *bioHx* structural gene were designed using the software Primer 5.0 ( http://www.premierbiosoft.com/). Restriction endonuclease *Bam*HI and *Hind*III digestion sites were designed and indicated by underlined sequences, respectively. PCR amplification was performed in a 20 μl reaction volume containing 1×PrimeSTAR® MAX Premix (TaKaRa), 5 μM each of primers, and approximately 10 ng template DNA. PCR reactions were carried out under the following condition according to the instructions of the supplier: 30 cycles consisting of 98°C for 10s, 55°C for 5 s, and 72°C for 10s; and a final extension of 72°C for 5 min. Amplification was performed in a Mastercycler® pro PCR thermal cycler (Eppendorf, Hamburg, Germany). A sample (5 μl) of each amplification reaction was analyzed by electrophoresis on a 1.0% agarose gel.

The desired amplicon was purified, and digested with the two restriction endonucleases (Promega) at 37°C according to the instructions of the supplier. Restriction fragment was then purified, and ligated into the expression vector pET-28a that was digested with the same endonucleases. Ligation DNA was transformed into *E. coli* BL21 competent cells by the heat-shock method. Transformants grown on LB-kanamycin agar plates were identified by colony PCR assay. Plasmid DNA of positive transformants were isolated and digested with *Bam*HI and *Hind*III. Recombinant plasmid, pET28a-*bioHx*, was used for protein expression analysis.

When *E. coli* BL21 carrying pET28a-*bioHx* was grown in LB-kanamycin broth at 37°C to OD_600_ 0.3-0.6, IPTG was added to a final concentration of 0.6 mM, and the cells were cultured at 21°C for additional 20 h. The cells were harvested by centrifugation at 10,000 g for 10 min at 4°C, and then resuspended in 5-fold of BugBuster protein extraction reagent (Merck Millipore), followed by incubation at 25°C for 20 min. The suspension was incubated on ice for 30 min, and then sonicated five times each for 3 sec. The cell lysate was centrifuged at 16,000 g for 20 min at 4°C, and the supernatant containing soluble target protein was collected and analyzed by one-dimensional sodium dodecyl sulfate-polyacrylamide gel electrophoresis (SDS-PAGE) with 12% separation gel and 5% stacking gel using a Mini-PROTEAN® electrophoresis cell (Bio-Rad). Following electrophoresis, the gel was stained with 0.25% Coomassie brilliant blue R250 and then destained according to the standard method [22].

The cell-free extract was purified using Ni-NTA His·Bind® resin (Merck Millipore) according to the supplier’s instructions. The resulting protein fractions were collected and analyzed by SDS-PAGE. Protein concentration was determined using BCA protein assay kit (Sangon) with bovine serum albumin as the standard. The *E. coli* BL21 carrying the vector pET28a was used as a control.

### Characterization of BioHx

Purified BioHx was subjected to several biochemical assays including substrate specificity, optimum temperature and pH, and effects of organic solvents, detergents and metal ions on enzyme stability. The data was expressed as an average of the results from triplicate assays. Carboxylesterase activity of BioHx was measured spectrophotometrically according to the method described by Min-A et al. [6], using *p*-nitrophenyl (*p*NP) butyrate (C4) or *p*NP esters of other fatty acids (C2–C12) (Sigma-Aldrich) as the substrates. Purified enzyme was appropriately diluted to 50 μl and incubated with a 200 μl reaction mixture containing 10 mM *p*-NP butyrate or other *p*NP esters, ethanol, and 50 mM Tris-HCl (pH 8.0) at 1:4:95 (by vol). The amount of *p*-nitrophenol released from *p*NP esters at 30°C was monitored at 405 nm for 10 min using a spectrophotometer Synergy™ 2 (BioTek). One unit of enzyme activity was defined as the amount of enzyme capable of releasing 1 μmol of p-nitrophenol esters per minute. Thioesterase activity was measured according to the method described by Watanabe et al. [26] using palmitoyl-CoA (ACP) as the substrate.

The optimum temperature for enzyme activity was determined at temperatures between 20 and 80°C at pH 8.0 using *p*-NP butyrate as the substrate. The thermostability of BioHx was assayed by incubating aliquots of purified BioHx at various temperatures (10 to 80°C ) for 30 min. The residual activities were measured at 30°C. The effect of pH on enzyme activity was investigated at 30°C at the pH range of 3.0 to 10.0, 50 mM sodium citrate was used for pH 3.0-6.0, 50 mM sodium phosphate for pH 6.0-8.0 and 50 mM Tris-HCl for pH 8.0-10.0 [6]. The pH stability of the enzyme was evaluated by incubating BioHx at various pH values (3.0-10.0) at 4°C for 12 h. The residual enzyme activity was determined under standard condition at 30°C and pH 8.0 using the *p*-NP butyrate substrate.

The stability of purified BioHx in the presence of metal ions (10 mM), detergents (0.25-0.5%) and organic solvents (20-50%) was determined by incubation of the enzyme with various reagents at 30°C for 1 h, residual enzyme activity was measured under the standard condition.

## Competing interests

The authors declare that they have no competing interests.

## Authors' contributions

YS, YP, BL and LC participated in the design of the study; YS carried out the major experiments, and HW carried out DNA sequencing; YS, YP, BL and LC analyzed data; LC drafted the manuscript, and QS revised it for important intellectual content and improvement; YS, YP, BL, HW, QS and LC read and approved the final manuscript to be published.
